# THE GRAYING RAINBOW: TRACING LGBTQI AGING IN SCANDINAVIAN LITERATURE

**DOI:** 10.1093/geroni/igad104.2475

**Published:** 2023-12-21

**Authors:** Mats Christiansen, Mika Handelsman-Nielsen, Manijeh Mehdiyar

**Affiliations:** Åbo Akademi University, Solna, Sweden; The Public Health Agency of Sweden, Solna, Stockholms Lan, Sweden; The Public Health Agency of Sweden, Solna, Stockholms Lan, Sweden

## Abstract

Background: There have been several international reviews about aging LGBTQI health and living conditions, but where policy, social insurance, and services differ. The Public Health Agency of Sweden was given a government assignment to review the literature on LGBTI, emphasizing Scandinavian literature. Method: This scoping review includes peer-reviewed literature published in English or Scandinavian languages from January 1, 2012, to May 2022. Literature was searched in PsycINFO, PubMed, Web of Science, CINAHL, International Bibliography of the Social Sciences (IBSS), Social Science Database, Applied Social Sciences Index & Abstracts (ASSIA), and Sociological Abstracts, Sociology Database. Initially, 11,428 articles were found. After removing duplicates using Rayyan and reviewing titles and abstracts, 54 articles were read in full. After the final review, 16 articles remained. Thematic analysis was used to produce themes from the reviewed literature. Findings: The following two themes were identified: Aspects of health and Living conditions. Studies were primarily interpretive. There is a lack of studies about some subgroups of older LGBTQ people in this context; for instance, there are insufficient studies on older lesbian women and gay men. Furthermore, there is a lack of studies on somatic health for older LGBTQ people, generally. Comparatively, albeit in small samples, we better understand trans descriptions of aging than lesbian women and gay men. There were no studies found on intersex individuals. Implications: There remains a paucity of literature regarding the life and living conditions for LGBTQI older adults in Nordic countries.

